# Language-based personality assessment from life narratives: a focus on model interpretability and efficiency

**DOI:** 10.3389/frai.2026.1760246

**Published:** 2026-05-08

**Authors:** Rasiq Hussain, Zerui Ma, Ritik Khandelwal, Joshua Oltmanns, Mehak Gupta

**Affiliations:** 1 Department of Computer Science, Southern Methodist University, Dallas, TX, United States; 2 Department of Psychological & Brain Sciences, Washington University, St. Louis, MO, United States

**Keywords:** attention mechanisms, Big Five personality, interpretability, language models, life narratives, natural language processing, personality assessment, transformer models

## Abstract

Natural Language Processing (NLP) enables novel approaches to personality assessment by analyzing rich, open-ended narratives, rather than relying solely on structured questionnaires. While most existing work focuses on short-form social media texts, this study shifts attention to predicting the Big Five personality traits, a framework closely tied to mental-health outcomes, from long-form life narrative interviews. Each narrative exceeds 2,000 words, posing significant challenges for standard language models. We propose a two-step modeling framework that captures contextual representations of long texts while prioritizing interpretability and computational efficiency. First, we extract contextual embeddings using a sliding-window finetuning strategy on pretrained transformer models. These embeddings are then processed with Recurrent Neural Networks (RNNs) equipped with attention mechanisms to capture long-range dependencies and provide interpretable outputs. Our hybrid approach effectively combines the representational power of transformers with the sequence-handling strengths of RNNs. Through comparisons with state-of-the-art long-context models and interpretability analyses, we demonstrate improvements in prediction accuracy, computational efficiency, and interpretability. These findings underscore the potential of interpretable and efficient language models for assessing personality from life narratives.

## 1 Introduction

Self-report instruments have long been the standard in psychological assessment due to their simplicity and ability to produce quantitative trait scores. However, they are highly dependent on individuals’ self-insight and are vulnerable to various forms of response bias. As a result, these tools are not always used consistently in clinical practice. In fact, only about half of psychologists report routinely using standardized assessments (Piotrowski, 1999; Soto, 2019; Wright et al., 2017). This has prompted growing interest in incorporating richer modalities—such as language-based tools—into psychological assessment frameworks (Paulhus and Vazire, 2007; Hatfield and Ogles, 2004; Wright et al., 2017; Kjell et al., 2022). The Big Five personality model (also known as the Five Factor Model) Costa and McCrae (2008) provides a strong foundation for such approaches, as its five domains—Openness, Conscientiousness, Extraversion, Agreeableness, and Neuroticism—can be reliably inferred from linguistic patterns in narrative text.

The Big Five is not only one of the most well-validated and widely used models in psychological science but is also closely tied to mental-health functioning. Its domains underlie the structure of mental disorders (Kotov et al., 2010, 2017), show longitudinal stability, and predict a range of mental-health and behavioral outcomes (John, 2021; Ozer and Benet-Martinez, 2006). Given its clinical relevance, the Big Five offers an ideal framework for evaluating language-based personality assessment tools, particularly when applied to rich, open-ended life narratives. While language-based tools hold significant promise in psychological assessment, their application to life narrative interviews presents unique challenges.

To the best of our knowledge, this is the first study to apply NLP-based personality prediction to life narrative interviews to predict Big Five personality scores. These interviews over 2000 tokens, demand models that can process long-form text without losing essential context. Models like BERT and RoBERTa, designed for shorter texts (up to 512 tokens), struggle to capture the full context of long interviews without significant adaptations (Cutler and Condon, 2023; Lynn et al., 2020; Pappagari et al., 2019). While newer models like Longformer and Llama extend token limits, they still face real-world constraints such as memory, training time, and efficiency (Zhang et al., 2020; Ding et al., 2020). Moreover, their lack of interpretability limits their clinical applicability.

To address the challenge of processing long-form narratives while preserving meaningful context and maintaining computational efficiency, we investigate how pre-trained language models (PLMs), such as RoBERTa-L, can be adapted to overcome token limitations (Zhang et al., 2020; Ding et al., 2020). In addition, we tackle the challenge of interpretability in large deep learning models by incorporating an attention mechanism, which allows for a clearer understanding of the model’s decision-making process.

## 2 Background and related work

### 2.1 Language and psychological assessment

The link between language and psychological phenomena has long been established (Boyd and Schwartz, 2021; Tausczik and Pennebaker, 2010). NLP and AI tools have been applied to understand identity (Argamon et al., 2007; Berger and Packard, 2022; Kwantes et al., 2016; Schwartz et al., 2013), emotion (De Bruyne et al., 2022; Eichstaedt et al., 2018; Sun et al., 2020), behavior (Curtis et al., 2018; Kjell et al., 2021; MacAvaney et al., 2021), and other psychological traits (Eichstaedt et al., 2021; Iliev et al., 2015; Jackson et al., 2022; Schwartz and Ungar, 2015). In particular, Big Five personality has been successfully detected through language (Park et al., 2015), which serves as the foundation for many personality assessment models in NLP.

### 2.2 Recent advances in personality prediction

Recent advancements in Natural Language Processing (NLP), particularly with deep learning and large language models (LLMs), have significantly improved personality prediction in a classification task (Jain et al., 2022; Mehta et al., 2020; Ganesan et al., 2023; Peters et al., 2024). Much of this progress has been demonstrated on short-form social media texts such as tweets, Facebook posts, and Instagram captions (Liao et al., 2021; Joshy and Sundar, 2022). Several studies have fine-tuned transformer-based models on social media data to predict personality as a continuous Big Five personality scores in a regression task (Cutler and Condon, 2023; Liu et al., 2019; Simchon et al., 2023; Ganesan et al., 2023).

More recent studies have explored the use of large language models and generative AI for personality inference. For example, Wright et al. (2026) demonstrate zero-shot generative AI scoring of brief openended text. Similarly, recent work shows that LLMs can infer psychological traits directly from social media posts without task-specific training (Peters et al., 2024). Other studies demonstrate that language model embeddings capture perceived personality signals of public figures (Cao and Kosinski, 2024), while deep learning approaches combining linguistic and acoustic signals have also been applied to predict personality traits from speech and conversational data (Lukac, 2024; Alsini et al., 2024). Additionally, emerging benchmarks have begun evaluating LLM-based personality inference on real-world conversational datasets paired with self-reported personality scores (Zhu et al., 2025). Together, these studies highlight the growing capability of modern language models to capture personality-related cues from natural language.

### 2.3 Challenges of long-context personality prediction

Most of these studies focus on short, discrete text samples, such as social media posts or survey responses. In contrast, life narrative interviews, as studied here, are continuous and long, often exceeding 2000 tokens, and require models capable of handling long-context dependencies while preserving subtle personality cues. While some work has used interview-style datasets such as DAIC-WOZ for tasks like depression detection (Danner et al., 2023; Senn et al., 2022), these scenarios often contain more explicit cues than the subtle signals present in open-ended life narratives.

Approaches like sliding windows (Wang et al., 2019) and hierarchical aggregation methods combine representations of smaller segments (Geng et al., 2024; Lynn et al., 2020). However, these approaches may lose important global context or struggle with gradient stability across long sequences. Other approaches, such as selecting emotional words from each sentence to reduce input length (Bajestani et al., 2024), simplify long texts but fail to capture the contextual dependencies necessary for interpreting nuanced personality traits.

### 2.4 Existing solutions and limitations

#### 2.4.1 Extended token limit models

Extended token limit models like Llama and Longformer (Beltagy et al., 2020; Zhang et al., 2020; Ding et al., 2020) offer potential solutions, but with high computational costs and slower training times. These models also struggle to effectively utilize information distributed across long sequences. This limitation is commonly referred to as the lost-in-the-middle phenomenon (An et al., 2024; Beltagy et al., 2020; Liu et al., 2024; Huang et al., 2023). In this scenario, models tend to focus on tokens at the beginning and end of long inputs while under-attending to information in the middle.

Such uneven attention can contribute to prediction instability, often described as over-drift, where early contextual signals gradually lose influence as later tokens dominate attention, potentially leading to biased inference in downstream tasks. Architectural surveys highlight that attention mechanisms and global context patterns are key contributors to this drift behavior (Huang et al., 2023). As a result, important evidence in the middle of long sequences may be overlooked, reducing long-context retention and retrieval fidelity, and sometimes leading to inconsistent model reasoning biased toward the sequence boundaries.

#### 2.4.2 Other approaches to long-context modeling

Recent work attempts to mitigate the issue of lost context through selective segment extraction or retrieval mechanisms, such as identifying salient passages (An et al., 2024) or selecting unique representative segments (Geng et al., 2024).

Other models, such as Transformer-XL (Dai et al., 2019) and Compressive Transformer (Rae et al., 2019), aim to address long-term dependencies through memory cells and history condensation. Models like Memformer (Wu et al., 2020) dynamically retrieve and update memory, while ERNIE-Doc (Ding et al., 2021) concatenates historical segments to the current context.

These architectural strategies have been proposed to preserve long-range contextual signals and reduce drift or information decay in long-sequence reasoning. However, even with these advances, maintaining stable utilization of extended context remains a challenging task (Huang et al., 2023).

To efficiently capture long-text context using models with fewer parameters and lower computational cost, Zhang et al. (2020) proposed BERT-AL, combining BERT with multi-channel LSTMs to summarize long text. Geng et al. (2024) handles long sequences by aggregating embeddings of smaller texts. Lynn et al. (2020) encode each social-media post using pre-trained BERT, where each post is smaller than the token limit of BERT, and apply a GRU with post-level attention over post representations to predict personality traits from Facebook data. Hierarchical approaches like those in Lynn et al. (2020), Pappagari et al. (2019), and Gong et al. (2020) combine transformer or recurrent layers over segment embeddings. We adopt a similar strategy but enhance it with two-step training and attention layers for model training stability and interpretability (Li et al., 2016), respectively.

### 2.5 This study: computationally efficient and interpretable modeling

While prior work has demonstrated personality prediction from social media or short text using LLMs and hybrid architectures, our study uniquely targets long life narratives with a computationally efficient and interpretable two-step framework. By combining segment-level fine-tuning with sequential aggregation, our framework extends existing approaches to efficiently model long-context narratives while maintaining interpretability and prediction stability.

Unlike prior work that uses hierarchical architectures to aggregate pre-trained language model embeddings from separate social media posts (Lynn et al., 2020; Pappagari et al., 2019; Gong et al., 2020), our approach captures the continuous context of long life narratives. To achieve this, we propose a computationally efficient and interpretable two-step architecture. In the first step, a small pretrained language model (RoBERTa-L) is fine-tuned on overlapping narrative segments using a sliding-window strategy to generate optimized segment-level embeddings. In the second step, these embeddings are sequentially aggregated using an RNN with attention to capture contextual dependencies across the narrative.

This design separates representation learning from sequence modeling, improving gradient stability and computational efficiency while preserving long-context information. The attention mechanism further enables trait-specific interpretability by highlighting narrative segments that contribute most to the predicted personality scores.

In this work, personality traits are predicted as continuous regression scores, reflecting the dimensional nature of the Big Five framework. By addressing the challenges of long-context modeling and interpretability in narrative data, our approach contributes a practical and scalable framework for computational personality assessment.

The main contributions of this paper can be listed as follows:

1. A two-step approach for personality prediction from long-form life narratives, leveraging finetuned RoBERTa-L for segment-level embeddings and RNN attention to preserve continuous context, improving accuracy and computational efficiency.
2. Interpretability through attention layers, which enables clinicians to understand the factors driving personality predictions.
3. A thorough empirical evaluation that demonstrates the performance, efficiency, and interpretability of our approach compared to other LLM models.

## 3 Materials and methods

### 3.1 Data

The dataset used in this study is derived from the St. Louis Personality and Aging Network (SPAN) and comprises life narrative interviews with 1,408 older adults aged 55–64 from the St. Louis area (Oltmanns et al., 2020). During the interviews, participants recount their lives in four chapters beginning at age 18, describing key events and influential individuals. These narratives provide rich linguistic data reflecting participants’ life experiences and align with psychological approaches that study personality through life stories.

Personality traits were measured using the Big Five framework, which models personality across five domains: Openness, Conscientiousness, Extraversion, Agreeableness, and Neuroticism (OCEAN). Participants completed the NEO Personality Inventory–Revised (NEO-PI-R; Costa and McCrae, 2008), a 240-item self-report questionnaire with responses rated on a Likert scale from 0 (strongly disagree) to 4 (strongly agree). Each personality domain is assessed using 48 items, yielding raw trait scores ranging from 0 to 192.

In this study, we use only the participant self-report NEO-PI-R domain scores as the ground-truth personality labels. Domain scores were computed following the SPAN study protocol. If one or two items were missing, the completed items were averaged to compute the domain score; participants missing more than two items were excluded from that scale. The NEO-PI-R has demonstrated strong psychometric properties in the SPAN dataset, including high internal consistency, test–retest reliability, and criterion validity across both self-reports and informant ratings (Oltmanns et al., 2020; Wright et al., 2022).

#### 3.1.1 Data preprocessing

The interview transcripts were preprocessed to remove non-content annotations and to separate interviewer and participant speech. On average, each transcript contained 2,513 words. From the original set of transcripts, we excluded 8 interviews containing fewer than 50 words, resulting in the final dataset used in our experiments.

The corresponding Big Five trait scores range from 0 to 192. To stabilize training and ensure comparable optimization across models, target values were standardized using z-score normalization implemented via the StandardScaler from the scikit-learn library. Specifically, trait scores were transformed as: $y_{\mathrm{norm}}=\frac{y-\mu }{\sigma },$ where *μ* and *σ* denote the mean and standard deviation of the training targets.

To prevent data leakage, the scaler was fit only on the training portion of each fold in the 5-fold crossvalidation and then applied to the corresponding test sets. Model predictions were generated and evaluated in the standardized (z-score normalized) space. All evaluation metrics (MSE and *R*^2^) are reported in this normalized space for consistency across models (see Table 1).

**Table 1:** Dataset statistics showing transcript lengths and Big Five personality score distribution.

Metric	Mean	Std	Min	Max
Transcript length (words)	2,513	1,142	50	8,247
Openness	115.2	18.4	65	168
Conscientiousness	128.7	16.9	78	175
Extraversion	118.3	19.2	62	172
Agreeableness	134.1	15.7	85	180
Neuroticism	89.4	21.3	38	156

### 3.2 Models and methods

Below we describe our strategies for handling long life narrative interviews using both small and large language models, followed by our proposed two-step approach for efficient, and interpretable modeling.

#### 3.2.1 Fine-tuning smaller pre-trained language models

We fine-tuned three transformer-based models—RoBERTa-L (355 M), XLM-RoBERTa-L (550 M), and XLNet (340 M)—to predict Big Five personality scores from narrative interviews. While these models offer strong contextual understanding, they are constrained by a 512-token input limit, whereas our average transcript length is approximately 2,992 tokens.

**Figure 1:**
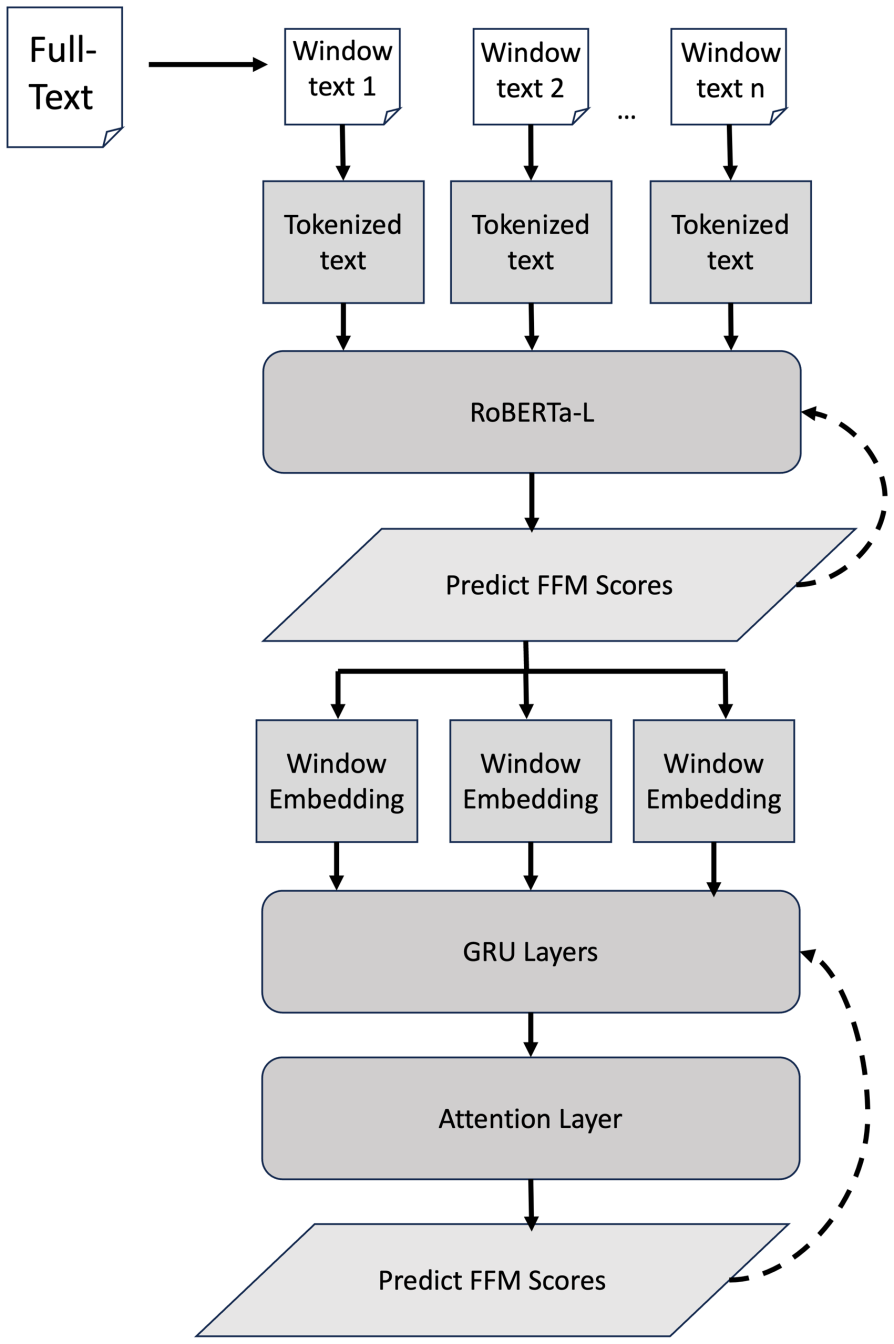
RoBERTa-L + RNN architecture. Dashed arrows show backpropagation with (1) fine-tuning RoBERTa-L with sliding-windows on Big Five personality scores and (2) training RNN with embeddings from fine-tuned RoBERTa-L.

To work within this limit, we employed a sliding-window approach, segmenting each transcript into overlapping 512-token chunks. Each model was fine-tuned on these chunks, and the final personality score was obtained by taking the median of window-level predictions. This method enables long text processing with smaller pre-trained language models (PLMs), but it treats windows independently and fails to capture global narrative coherence (see Figure 1).

#### 3.2.2 Fine-tuning large language models

To effectively process long life narrative interviews, we fine-tuned two large language models (LLMs): Longformer and LLaMA 3.2 1B, chosen for their capacity to handle extended contexts.

Longformer, with 435 million parameters, employs sparse attention mechanism to efficiently process sequences up to 4,096 tokens without overlapping windows. Unlike standard transformers, where every token attends to every other token leading to quadratic complexity (*O*(*n*^2^)), Longformer restricts attention to local windows for most tokens, which captures nearby context with low computational cost. To retain important global information, a small subset of global tokens can attend to all positions, enabling the model to propagate information across distant parts of the sequence. This design reduces memory and computation requirements while still preserving essential long-range dependencies. However, because most attention is local, fully capturing dependencies across the entire narrative can be challenging, which may limit performance on tasks requiring holistic document understanding.

LLaMA 3.2 1B, an autoregressive transformer can process up to 128 K token, in a single forward pass. While this enables retention of long-range dependencies and global context, the quadratic scaling due to token-to-token attention in long input length imposes high resource demands for fine-tuning. To address this, we applied LoRA (Low-Rank Adaptation), a parameter-efficient fine-tuning technique that updates only a small subset of model weights, and quantized the model to bfloat16 to reduce memory usage.

These models illustrate complementary architectural strategies for handling long narratives. Longformer provides computational efficiency for long sequences through sparse attention, capturing local context and selective global information. LLaMA 3.2 1B supports full narrative processing with extended context, with parameter-efficient fine-tuning and quantization used to manage resource requirements. Together, these approaches reflect the trade-offs between sequence length, memory efficiency, and long-context reasoning, highlighting the challenges of scaling LLMs for long-text interviews. To address these challenges while maintaining full-context understanding and reducing computational overhead, we propose a hybrid approach combining smaller PLMs with recurrent networks.

#### 3.2.3 Language model and recurrent network

To efficiently capture the full-context of long text while taking advantage of contextual language understanding of PLMs without increasing computational and memory cost we combine smaller PLMs (RoBERTa-L) with recurrent neural network (RNNs). This is done using a two-step approach:

First, RoBERTa-L is fine-tuned via a sliding-window strategy to predict Big Five personality scores. Given a set of input transcripts *X* = {*X*_1_*,…, X_i_,…, X_N_*}, we apply a sliding window of size *w* and stride *s*, resulting in a total number of windows (T) for a transcript X_i_ of length L_i_ as defined below in Equation 1:


$$T=\frac{L_{i}-w}{s}+1$$ (1)

We fine-tune the transformer model T(X_i_) (RoBERTa-L in our implementation) using mean squared error (MSE) loss to predict Big Five personality scores. The fine-tuned T(X_i_) is then frozen and used to extract [CLS] embeddings from each window of transcript *X_i_*:


$$(\mathrm{CL}S_{1},\ldots ,\mathrm{CL}S_{T})=T\;{\left(X_{i}\right)}_{\left[\mathrm{CLS}\right]}$$ (2)

Second, the extracted embeddings (*CLS*_1_*,…, CLS_T_*) are sequenced and passed through a two-layer GRU (a variant of RNN) with a hidden size of 256 (Equation 2). The model is trained using mean squared error (MSE) loss to predict Big Five personality scores. During GRU training, gradients are propagated only through the GRU layers, preventing updates to RoBERTa-L and thereby reducing residual error from the base encoder.

The GRU processes the sequence of [CLS] embeddings and produces a sequence of hidden states {*h*_1_*,…,h_T_*}, where each *h_t_* represents the hidden state for the *t*-th window:


$$\left\{h_{1},\ldots ,h_{T}\}=\mathrm{RNN}(\mathrm{CL}S_{1},\ldots ,\mathrm{CL}S_{T}\right)$$ (3)

To obtain a context vector *c_i_*, attention is applied over the hidden states (Equation 3). The normalized attention weights *α_t_* are computed as:


$$a_{t}=\frac{\exp(w^{T}h_{t})}{\sum_{k=1}^{T}\exp\;(w^{T}h_{k})}$$ (4)

Attention score *α_t_* highlights key text windows of a transcript in predicting Big Five personality scores.

Using these weights, the context vector *c_i_* is calculated as the weighted sum of hidden states:


$$c_{i}=\sum_{t=1}^{T}\alpha_{t}\cdot h_{t}$$ (5)

The context vector *c_i_* is fed into a fully connected layer to predict Big Five personality scores, enabling long-term context modeling with focused attention on important text windows. The learned attention weights *α_t_* not only improve prediction by emphasizing relevant parts of the narrative but also provide interpretability by highlighting which windows contribute most to the final personality estimate.

### 3.3 Experiment design

All models were trained using 5-fold cross-validation, resulting in an 80:20 train:test split for each of the five runs. 5% of the training set was reserved as a validation set for early stopping. The splits were kept consistent across both stages of the two-step method within each fold. For a given fold, RoBERTa-L was fine-tuned exclusively on the training portion of that fold and subsequently frozen to generate embeddings for the corresponding training, validation, and test sets within the same fold. To prevent data leakage, transcripts were assigned to a single fold only, ensuring that no transcript or participant appeared in multiple folds.

Models were trained for up to 50 epochs with early stopping (patience = 5) based on validation MSE, and the best-performing checkpoint was retained. Test performance was assessed using mean squared error (MSE) and *R*^2^ score.

For all experiments, each Big Five trait is predicted using an independent single-task model.

Specifically, we train five separate models—one for each trait (Openness, Conscientiousness, Extraversion, Agreeableness, and Neuroticism). Attention weights are computed separately for each model, enabling trait-specific interpretability, which supports the analysis of attention patterns for individual personality traits.

We conduct comprehensive experiments comparing our proposed model against five baselines of existing language models, as well as five ablations of our own architecture. These comparisons are designed to assess the impact of fine-tuning, architectural choices, and training strategies on model performance.

#### 3.3.1 Baseline models

For baseline comparison, we compare our model with existing smaller PLMs and LLMs. The list of PLMs includes: (1) RoBERTa-L: A larger variant of RoBERTa, (2) XLM-RoBERTa-L: A cross-lingual model based on RoBERTa, and (3) XLNet-L: A variant of XLNet, a pre-trained autoregressive model. The list of LLMs includes: (1) Longformer: A model designed to handle long sequences, and (2) LLaMA 3.2 1B: A 1 billion parameter variant of the LLaMA language model.

#### 3.3.2 Ablation components

We evaluate the effectiveness of our proposed two-step training strategy by comparing it with a continuous flow (CF) hierarchical setup, where RoBERTa-L window embeddings are aggregated using either an RNN or Transformer. In the CF approach, gradients from the downstream sequence model (either an RNN or Transformer) are backpropagated into the RoBERTa-L model, allowing end-to-end fine-tuning of both components. This setup directly implements the continuous flow hierarchical architecture (RoBERTa-window embeddings + sequential aggregator trained jointly), providing a rigorous comparison for our two-step method. We also test variation of the two-step strategy, including using a Feed-Forward Network (FFN) as the aggregator and using pretrained RoBERTa-L embeddings instead of fine-tuned embeddings, to isolate the effect of sequential aggregation and fine-tuning.

We evaluate the following five variants: (1) FT-RoBERTa-L + RNN CF: RNN with CF gradients backpropagated into fine-tuned RoBERTa-L, (2) FT-RoBERTa-L + Transformer CF: Transformer with CF gradients backpropagated into fine-tuned RoBERTa-L, (3) PT-RoBERTa-L + RNN 2-step: Pretrained RoBERTa-L embeddings with RNN in the two-step framework, (4) FT-RoBERTa-L + FFN 2-step: Feedforward network in the two-step framework, and (5) FT-RoBERTa-L + Transformer 2-step: Fine-tuned RoBERTa-L with Transformer in the two-step framework.

#### 3.3.3 Implementation details

##### 3.3.3.1 PLM baselines

All pretrained language models (PLMs) - RoBERTa-L, XLM-RoBERTa-L, and XLNet-L were initialized from Hugging Face checkpoints and fine-tuned for regression using the simpletransformers library and were trained using the Adam optimizer with a learning rate of 2 × 10^−5^ and batch sizes of 8. We used a maximum window size of 512 tokens with a stride of 256 tokens (50% overlap). This produced approximately 15 windows on average and at most about 37 windows for the longest transcripts. For compatibility with the downstream sequential model, window sequences were padded to a fixed maximum length of 40 segments. Any partial last window that does not fill 512 tokens is included and right-padded with zeros.

##### 3.3.3.2 LLaMA

A single-layer perceptron (SLP) was added for regression to predict Big Five personality scores directly from narrative input. To reduce the significant computational demands of full fine-tuning LLaMA, we applied parameter-efficient fine-tuning (PEFT) using the LoRA method via the SFTTrainer. We configured LoRA with a rank *r* = 64, a scaling factor *lora_α_* = 16, applied to the q proj, k proj, v proj, and o proj modules. The model was quantized to bfloat16 and trained using a learning rate of 2 × 10^−5^ and a batch size of 1 due to scaling limitation. Training was distributed across six NVIDIA A100 GPUs (80 GB VRAM each).

##### 3.3.3.3 Ablations

The Transformer model in both CF and 2-step approach used a same custom architecture consisting of 2 transformer layers with 8 attention heads and a 2048-dimensional feed-forward network. A dropout rate of 0.1 was applied after the self-attention layer, the feed-forward layer, and before the output layer. The RNN model in both CF and 2-step approach consisted of 2 layers of GRU. For the feed-forward network (FFN) variant in the two-step setup, we used two hidden layers with sizes 256 and 128.

All ablation variants, and the proposed framework, were trained using the same Adam optimizer with a learning rate of 1 × 10^−5^ and a batch size of 8 to ensure a controlled comparison and isolate the impact of architectural differences.

## 4 Results

### 4.1 Baseline comparison

Table 2 compares the performance of various pre-trained language models (PLMs), large language models (LLMs), and our proposed RoBERTa-L + RNN method for predicting personality scores. The FT-RoBERTa-L + RNN model achieved the best results, providing the lowest MSE and highest *R*^2^ across all personality traits. For example, it achieved an MSE of 0.69 (±0.01) and *R*^2^ of 0.30 (±0.01) for Openness, outperforming all other models.

**Table 2:** Comparison of mean MSE and *R*^2^ with standard deviation over 5-fold cross-validation between all baselines and our proposed model.

Model	Metric	O	C	E	A	N
RoBERTa-L	MSE	0.81_0.01_	0.98_0.05_	0.88_0.03_	0.96_0.03_	0.81_0.04_
	*R* ^2^	0.19_0.01_	0.02_0.05_	0.12_0.03_	0.04_0.03_	0.19_0.04_
XLM-RoBERTa-L	MSE	0.81_0.02_	0.92_0.04_	0.88_0.03_	0.93_0.02_	0.90_0.04_
	*R* ^2^	0.19_0.02_	0.03_0.05_	0.12_0.04_	0.07_0.02_	0.10_0.04_
XLNet-L	MSE	0.83_0.01_	0.99_0.05_	0.90_0.04_	0.89_0.03_	0.81_0.04_
	*R* ^2^	0.17_0.01_	0.01_0.05_	0.10_0.04_	0.03_0.02_	0.19_0.03_
Longformer	MSE	0.82_0.03_	0.97_0.05_	0.91_0.04_	0.96_0.04_	0.91_0.04_
	*R* ^2^	0.18_0.03_	0.03_0.05_	0.09_0.04_	0.04_0.04_	0.10_0.04_
Llama3.2 1B	MSE	0.74_0.02_	0.91_0.02_	0.99_0.02_	0.90_0.01_	0.96_0.02_
	*R* ^2^	0.25_0.01_	0.03_0.02_	0.05_0.02_	0.05_0.01_	0.01_0.03_
FT-RoBERTa-L+RNN	MSE	0.69_0.01_	0.68_0.05_	0.46_0.03_	0.56_0.02_	0.47_0.02_
	*R* ^2^	0.30_0.01_	0.30_0.05_	0.50_0.03_	0.43_0.02_	0.52_0.02_

In contrast, models like RoBERTa-L, XLM-RoBERTa-L, and XLNet-L exhibited moderate performance, with MSEs ranging from 0.81 to 0.96, but had low *R*^2^ values, indicating limited predictive power. The Longformer and LLaMA 3.2 1B models, despite their ability to process long sequences, performed similarly, with MSEs around 0.84 and *R*^2^ values not exceeding 0.25.

LLaMA 3.2 1B model, in particular, faced significant computational challenges due to its large parameter size and high memory demands. Despite its ability to process long sequences, LLaMA struggled with overfitting, showing a low training loss of approximately 0.02, but the validation loss remained high, fluctuating between 0.7 to 1.1.

### 4.2 Ablation analysis

As shown in Table 3, the variant using pre-trained RoBERTa embeddings with an RNN performs worst among all configurations, highlighting the importance of fine-tuning embeddings for task-specific adaptation. Comparing continuous flow (CF) variants with the proposed two-step approach, we observe that CF models underperform, suggesting that jointly training RoBERTa-L with the downstream sequence model may hinder convergence. Among the sequence models in 2-step framework, the FFN yields the lowest performance, indicating that ignoring sequential dependencies limits predictive accuracy. Transformers do not provide a clear advantage in this setting, likely due to the relatively short sequence length (≤ 40) In contrast, RNNs achieve a favorable balance between accuracy, interpretability, and computational efficiency. Our proposed two-step model achieves the highest *R*^2^ across all traits, outperforming all ablation variants.

**Table 3:** Ablation study results comparing different architectural choices and training strategies.

Model	Metric	O	C	E	A	N
FT-RoBERTa-L+RNN CF	MSE	0.96_0.02_	0.99_0.05_	1.50_0.03_	0.90_0.01_	1.10_0.03_
	*R* ^2^	0.03_0.01_	0.007_0.03_	0.02_0.02_	0.05_0.01_	0.01_0.03_
FT-RoBERTa-L+Transformer CF	MSE	0.94_0.02_	0.86_0.03_	0.96_0.03_	0.94_0.02_	0.89_0.03_
	*R* ^2^	0.04_0.02_	0.03_0.04_	0.05_0.02_	0.05_0.02_	0.01_0.03_
PT-RoBERTa-L+RNN 2-step	MSE	0.96_0.01_	1.01_0.04_	1.90_0.04_	1.12_0.02_	1.20_0.02_
	*R* ^2^	0.03_0.01_	0.001_0.04_	0.01_0.03_	0.02_0.01_	0.01_0.01_
FT-RoBERTa-L+FFN 2-step	MSE	0.82_0.02_	0.84_0.04_	0.81_0.03_	0.88_0.02_	0.81_0.02_
	*R* ^2^	0.12_0.01_	0.07_0.04_	0.17_0.02_	0.06_0.01_	0.15_0.01_
FT-RoBERTa-L+Transformer 2-step	MSE	0.75_0.02_	0.82_0.03_	0.70_0.02_	0.80_0.02_	0.79_0.03_
	*R* ^2^	0.20_0.02_	0.10_0.04_	0.20_0.02_	0.10_0.02_	0.18_0.02_
FT-RoBERTa-L+RNN 2-step	MSE	0.69_0.01_	0.68_0.05_	0.46_0.03_	0.56_0.02_	0.47_0.02_
	*R* ^2^	0.30_0.01_	0.30_0.05_	0.50_0.03_	0.43_0.02_	0.52_0.02_

### 4.3 Comparison of time and resource efficiency

We evaluate training complexity and inference performance of baseline models in Table 4, reporting parameter count, training time, GPU memory usage, estimated FLOPs, and inference latency. All models were fine-tuned with a batch size of 8 on NVIDIA A100 GPUs for fair comparison. Among models with the standard 512-token limit, RoBERTa-L is the most efficient, offering low training time, GPU memory usage, and FLOPs and XLNet-L require moderate increases in resources due to larger parameter count.

**Table 4:** Comparison of training times, model sizes and TFLOPs for different fine-tuned models.

Model	#Parameters	Train time	GPU (GB)	TFLOPs	Inference time (ms)
Roberta-L	335 mil	5:42	13	2.2	45
XLM-RoBERTa-L	550 mil	6:28	20	3.4	60
XLNet-L	340 mil	5:40	15	2.1	48
Longformer	435 mil	8:04	17	1.8	120
Llama-3.2-1B	1 billion	8:30	80	90	950

Models designed to handle longer contexts, such as Longformer and LLaMA-3.2-1B, incur substantially higher computational costs. LLaMA-3.2-1B, with 1 billion parameters, supports sequences up to 128 K tokens but requires high memory and compute because self-attention scales quadratically with input length. Its training requires 80 GB of GPU memory per batch and an estimated 90 TFLOPs per sample, nearly 40 × the FLOPs of RoBERTa-L. To accommodate long sequences, batch size was limited to 1, illustrating the scalability limits of large LLMs. Inference latency also scales with model size and input length. RoBERTa-L processes a 512-token sample in 45 ms, whereas LLaMA-3.2-1B requires 950 ms for a comparable input. These measurements highlight the trade-offs between long-context modeling, computational complexity, and real-time deployment feasibility.

The computational implications of model size extend beyond raw parameter counts. Large LLMs, such as LLaMA-3.2-1B, are most practical for centralized deployment on high-performance GPUs or TPUs due to their substantial memory and FLOPs requirements, which make edge or on-device deployment infeasible (Brown et al., 2020). Inference cost scales with user volume and usage intensity, rendering high-throughput applications resource-intensive. Parameter-efficient fine-tuning methods, such as LoRA (Hu et al., 2021), combined with mixed-precision training (e.g., bfloat16), can partially reduce these requirements, yet significant compute resources are still necessary for training and inference at scale. These factors directly affect MLOps pipelines, including distributed training, GPU scheduling, memory monitoring, and validation of throughput thresholds (Sendas and Rajale, 2024). In scenarios where deployment is cost- or resource-constrained, smaller PLMs or hybrid architectures that combine PLMs with lightweight sequential models (e.g., FT-RoBERTa-L + RNN) provide a practical and scalable alternative, efficiently handling long-text inputs without the prohibitive memory and computational overhead of very large LLMs.

### 4.4 Embedding visualization

**Figure 2:**
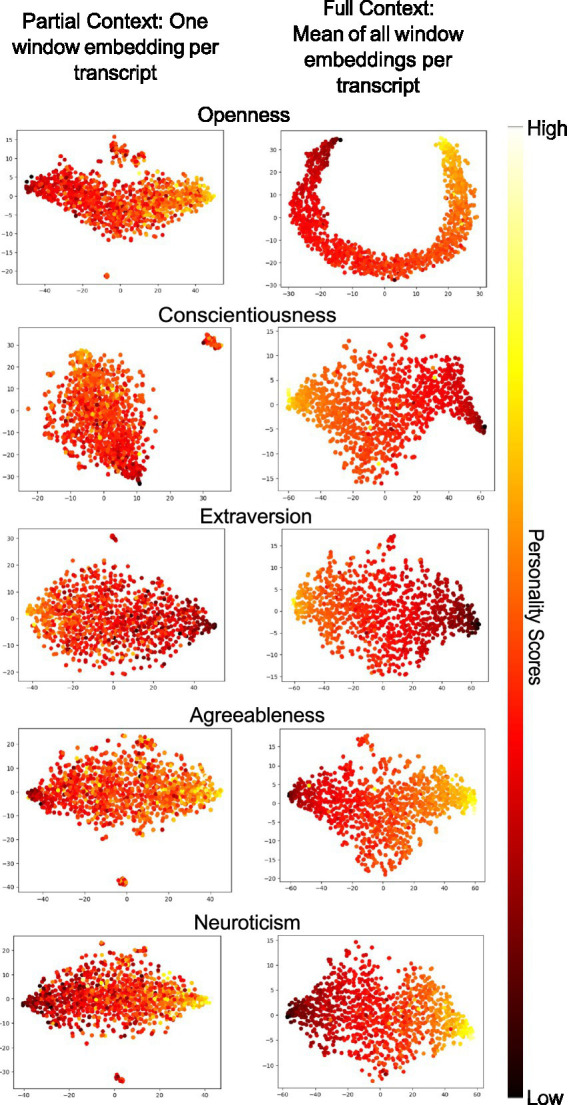
2-D visualization of [CLS] embeddings of training set from last layer of fine-tuned RoBERTa-L. The left panel shows embedding from a randomly chosen window per transcript, and the right panel shows the mean of all window-level embeddings. Ridge regression *R*^2^ values are displayed at the top of each image.

To understand the benefit of capturing full context versus partial context in life narrative interviews, we visualize high-dimensional text embeddings from the training dataset using t-SNE. Figure 2 presents two heatmaps: the left panel shows [CLS] embeddings from a single randomly selected window per sample, while the right panel shows the mean [CLS] embedding across all windows for each sample.

The left panel (single-window embeddings) shows little to no separation between high and low personality scores, suggesting that partial context from a single window lacks sufficient information for accurate prediction. In contrast, the right panel (mean embeddings across all windows) reveals a clearer gradient from dark to light, indicating better alignment with ground-truth scores. These results highlight the importance of full-context modeling.

### 4.5 Sensitivity and bias analysis

#### 4.5.1 Transcript length sensitivity

To examine sensitivity to transcript length and evaluate potential over-drift under varying context availability, transcripts were divided into three groups: Short (less than 1,500 words; 352 transcripts), Medium (1,500–3,500 words; 705 transcripts), and Long (greater than 3,500 words; 352 transcripts). This grouping allows us to assess both data-scarce scenarios (short transcripts) and extended-context scenarios (long transcripts).

Table 5 reports the MSE and *R*^2^ scores across these transcript length groups. Performance decreases for very short transcripts (*<*1,500 words), reflecting data scarcity where limited narrative information provides insufficient behavioral cues for reliable personality prediction.

**Table 5:** Performance across transcript lengths.

Dataset	Number	Metric	O	C	E	A	N
Full Dataset	1,409	MSE	0.69_0.01_	0.68_0.05_	0.46_0.03_	0.56_0.02_	0.47_0.02_
		*R* ^2^	0.30_0.01_	0.30_0.05_	0.50_0.03_	0.43_0.02_	0.52_0.02_
Short (*<*1,500 words)	340	MSE	0.59_0.02_	0.75_0.04_	0.58_0.03_	0.59_0.02_	0.40_0.02_
		*R* ^2^	0.24_0.02_	0.24_0.03_	0.35_0.02_	0.41_0.02_	0.50_0.02_
Medium (1500–3,500 words)	719	MSE	0.75_0.03_	0.73_0.03_	0.41_0.02_	0.51_0.02_	0.47_0.01_
		*R* ^2^	0.31_0.02_	0.34_0.02_	0.59_0.02_	0.51_0.01_	0.58_0.01_
Long (*>*3,500 words)	350	MSE	0.86_0.04_	0.57_0.03_	0.46_0.02_	0.55_0.02_	0.45_0.01_
		*R* ^2^	0.28_0.02_	0.23_0.02_	0.62_0.02_	0.46_0.02_	0.56_0.01_

Results report mean ± standard deviation across 5-fold cross-validation. The column Number indicates the number of transcripts in each group.

For medium-length transcripts (1500–3,500 words), performance improves and is often comparable to or slightly better than the full dataset, suggesting that moderate-length narratives provide a balance between sufficient behavioral evidence and manageable sequence length.

For long transcripts (*>*3,500 words), performance remains stable relative to the full dataset, indicating that extended context does not introduce instability or over-drift. In particular, Extraversion benefits from longer narratives, achieving an *R*^2^ of 0.62, demonstrating that the model can effectively leverage additional context without over-drift.

Across all transcript lengths, low standard deviations across folds indicate consistent and stable model behavior. These results collectively demonstrate that the proposed architecture effectively extracts traitrelevant signals from both moderate and extended narrative contexts, mitigating the risk of over-drift, while showing some susceptibility to data scarcity in very short sequences.

To further assess sensitivity to transcript length, we analyzed the correlation between transcript length and model predictions. Across all five OCEAN traits, correlations were not statistically significant (average *p*-value = 0.5), indicating that predictions do not systematically vary with input length. Together, these results demonstrate that the proposed FT-RoBERTa-L + RNN (2-step) architecture effectively extracts traitrelevant signals, mitigates over-drift, and is robust to variations in context length, while slight reductions in performance for very short transcripts can be attributed to data scarcity, reflecting limited behavioral cues in short sequences rather than instability.

#### 4.5.2 Gender bias analysis

We determine the Pearson correlation between gender and the predicted output for each model. The findings show that the relationship between gender and predicted values was not statistically significant in four of the five models (C, E, A, and N). However, a weak but statistically significant correlation was observed in the model for Openness (*r* = 0.1343, *p* = 0.0276), indicating a slight bias associated with this trait and gender.

### 4.6 Model interpretability

One of the key aspects of this study was about the RNN model’s interpretability. We have validated high-attention text windows with domain experts to ensure reliable predictions.

#### 4.6.1 RNN attention interpretability

**Figure 3:**
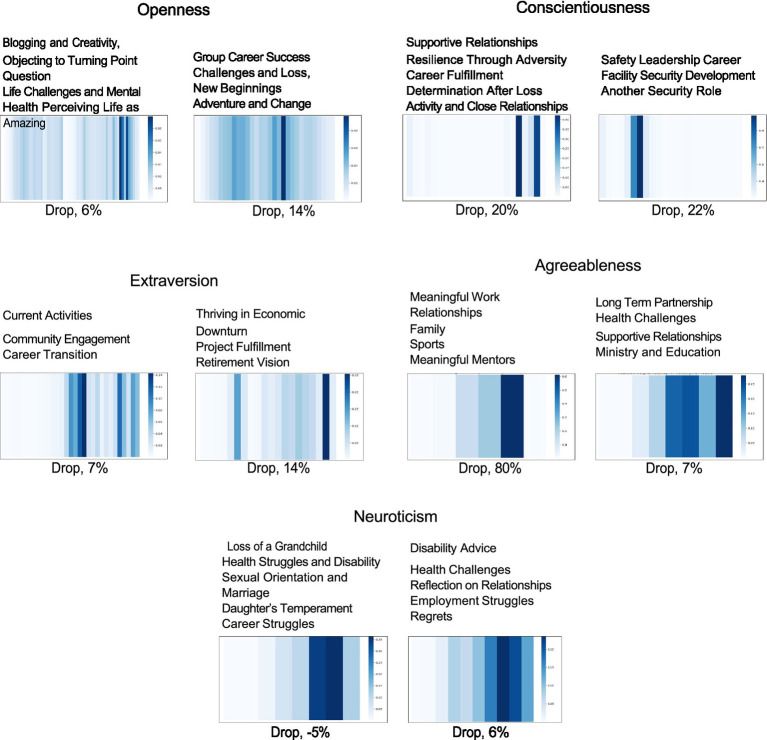
Attention distribution across text windows for two randomly selected examples from the top 10 highest predicted scores for each trait. For each example, the topics discussed in the three most highly attended text windows are listed at the top. The percentage shown below indicates the drop in the predicted score when the window with highest attention is removed.

Using attention mechanisms, the model focused on the transcript windows most relevant to each Big Five personality trait. For each trait, we randomly selected two examples from the top-10 transcripts in test set based on predicted scores and visualized the top attention windows as heatmaps in Figure 3.

For openness, high-attention windows included creative expression (photography, blogging), adventure, and excitement for career shifts in later life, in line with people who are more creative and open to experience (Schwaba, 2019). Conscientiousness examples emphasized career passion, responsibility, and drive, showing goal-oriented and achievement-focused individuals (Jackson et al., 2010). For extraversion, participants share active, socially engaged retirements, upbeat language about thriving through an economic downturn, and community involvement, reflecting traits of positivity and sociability (?). Agreeableness examples highlighted long-term relationships, mentorship, gratitude, and emotional warmth—core features of highly agreeable individuals (Graziano and Tobin, 2017). Finally, neuroticism examples reflected emotional struggles, health-related challenges, job loss, and social isolation, commonly associated with high neuroticism (Widiger and Oltmanns, 2017).

#### 4.6.2 Impact of high attention window-text on predicted score

We assess the impact of highly attended window-text on the predicted personality score. The highest attention window-text was identified and removed from the transcripts shown in Figure 3, and the model was evaluated both before and after this modification. The results indicated a noticeable decrease in the predicted scores for most transcripts when the highest attention window was excluded.

Score drops of 6 and 14% for Openness, 7% Extraversion (left), and 7% Agreeableness (right) were observed. In contrast, drops of 20 and 22% for Conscientiousness and 80% for Agreeableness (left).

were larger due to more concentrated attention. For Neuroticism, one transcript showed a 5% increase after removal, as the excluded window referenced overcoming loss, shifting focus to unresolved negative experiences.

#### 4.6.3 Context anywhere

**Figure 4:**
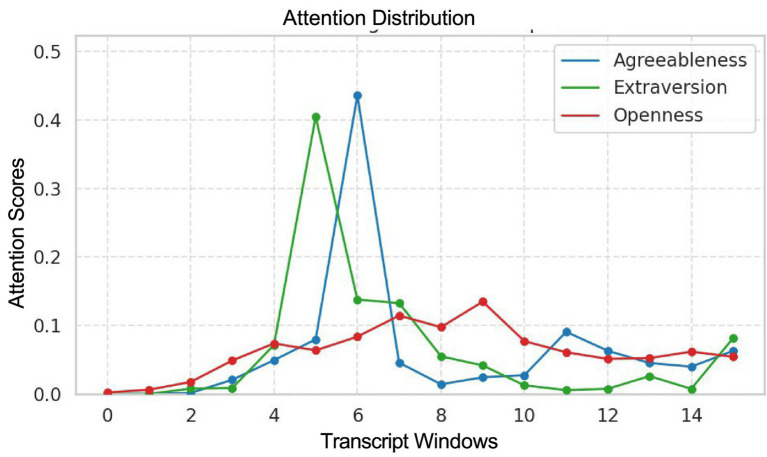
The plot shows the distribution of normalized attention scores across different transcript windows for predicting Agreeableness, Extraversion, and Openness. The *x*-axis represents transcript windows, and the *y*-axis represents attention scores. The figure illustrates that different personality traits exhibit distinct attention patterns, indicating that the model attends to different parts of the transcript when identifying trait-specific evidence.

In Figure 4, we analyzed a transcript that received 89, 112, and 96 scores for Agreeableness, Extraversion, and Openness, respectively. We examined the corresponding attention weight distributions produced by the RNN. As described in Equations 4, 5, the attention score *α_t_* represents the relative importance of each transcript window when constructing the context vector *c_t_* used for personality prediction. Higher attention values indicate segments that contribute more strongly to the final prediction. We normalize these attention scores to the sum of attentions scores across all transcript windows sum to 1. By comparing these normalized attention scores across traits (Agreeableness, Extraversion, and Openness), we observe that different portions of the transcript receive higher weights depending on the trait being evaluated, suggesting that the model selectively focuses on trait-relevant linguistic cues appearing throughout the transcript. Rather than concentrating attention only at the beginning or end of the text, meaningful signal appears across multiple segments of the transcript. This distributed weighting allows the model to incorporate evidence from across the transcript, which is particularly important for long-text scenarios and may improve prediction stability in practical deployment settings where relevant cues can appear at any point in a conversation.

#### 4.6.4 BERTopic modeling

**Figure 5:**
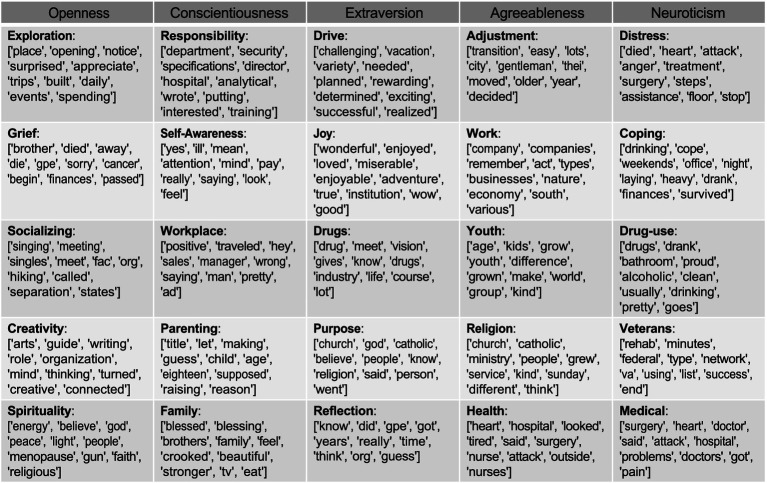
Top 5 correlated topics extracted from top-5 attention windows of test samples.

To further evaluate the effectiveness of RNN attention, we extract the top 5 correlated topics for each trait using BERTopic (Figure 5). These topics were generated using text from only the top five attention-weighted windows and not from the entire transcript.

The extracted topics reveal clear and meaningful patterns. Openness is strongly linked with topics like Exploration, Socializing, Creativity, and Spirituality. Conscientiousness aligns with structured domains—reflected in terms like” department,” “director,” “specifications,” and” training.” High Extraversion corresponds with socially active and energetic terms such as” vacation,” “adventure,” and” belief.” Agreeableness appears in prosocial and communal language within topics like adjustment, youth, and health. In contrast, Neuroticism is linked to distress-oriented themes, such as coping, drug use, veterans’ rehab, and medical hardship.

## 5 Discussion

Our two-step approach using RoBERTa-L with lightweight sequential modeling provides a computationally efficient solution for long-text prediction while effectively capturing trait-relevant signals across extended narratives. This study demonstrate the effectiveness of the proposed method, particularly the advantage of decoupling representation learning from sequence modeling. The CF hierarchical ablation comparison demonstrates the benefit of decoupling in the two-step approach. Fine-tuning RoBERTa-L independently on overlapping segments allows the model to better adapt to domain-specific language, resulting in optimized segment-level embeddings that capture the nuanced personality traits across the narrative. These highquality, context-sensitive embeddings enhance the performance of downstream models, contributing to improved personality prediction accuracy compared to the other models evaluated in this study.

Though we do not have a direct comparison in existing personality prediction studies that have used life narratives, for the purpose of comparison, existing work such as Piastra and Catellani (2025) used long essays for personality prediction and achieved a Pearson *R* of 0.28, compared to our value of 0.7. Other studies, including Danner et al. (2023), Senn et al. (2022), and Piastra and Catellani (2025), focused on classification rather than numerical prediction, which limits their applicability in real-world clinical scenarios.

While Longformer and LLaMA handle longer texts in a single pass, they involve key limitations. Prior work shows that Longformer’s sparse and global attention mechanisms influence how information flows across long sequences (Beltagy et al., 2020). More broadly, transformer architectures such as LLaMA processing extended contexts can suffer from degraded context utilization, including the “lost-in-the-middle” phenomenon, where mid-sequence information receives less attention (An et al., 2024). Architectural analyses further highlight trade-offs between attention complexity and long-context retention that may affect model stability and robustness (Huang et al., 2023). Consistent with these observations, long-context modeling remains challenging even for modern architectures. However, our transcript-length sensitivity analysis shows that the our proposed method maintains stable performance across medium and long transcripts without evidence of significant over-drift.

Moreover, our attention-based interpretability analysis shows that text in high-attention windows aligns closely with personality attributes reported in the literature. This supports model validation and enabled expert review, promoting trust in clinical applications. Overall, the two-step RoBERTa-L + RNN achieves stronger *R*^2^ while remaining substantially more efficient and interpretable than long-context LLMs, indicating a more practical route for language-based assessment from extended narratives.

## 6 Conclusion

Our study demonstrates that a two-step approach: fine-tuning a compact language model followed by a lightweight contextual aggregator, can outperform larger transformer-based models in both prediction accuracy and training efficiency. While applied here to personality prediction, the use of the Big Five framework, which is strongly connected to mental-health outcomes—underscores the relevance of our approach for mental-health applications. This interpretable and scalable method can also be broadly applied to other tasks requiring long-text processing using resource-efficient and transparent models.

## 7 Limitations and future work

Our work focuses exclusively on fine-tuning models for regression-based personality prediction and does not evaluate performance on generative tasks. Consequently, issues commonly associated with generative language models, such as hallucination or generative bias, were not examined. Future research can address this limitation by extending the framework to generative or hybrid settings. Incorporating strategies such as retrieval augmentation, adaptive context selection or fine-tuning before generative tasks could further improve context utilization in extended narratives and enhance our evaluation pipeline.

## 8 Ethical considerations

This study focuses on automated personality assessment from narrative interviews for research purposes. The framework emphasizes efficiency, and interpretability in personality prediction. All transcripts used in this work are anonymized and derived from participants who provided informed consent. The proposed approach is intended to support analysis of narrative patterns and cognitive traits rather than replace human judgment in high-stakes decision-making contexts.

Large language models may inherit biases from the data on which they are trained, potentially influencing downstream predictions. In the context of personality analysis, linguistic patterns associated with demographic or cultural factors may unintentionally affect model outputs. Careful dataset curation, fairness evaluation, and transparent reporting of model behavior are therefore important to mitigate bias in real-world deployments, particularly where automated assessments could influence decisions related to hiring, evaluation, or psychological profiling.

Another known limitation of large language models is hallucination, where models may produce plausible but incorrect outputs. Although the present work focuses on fine-tuning language models for structured personality prediction rather than open-ended generation, hallucination risks remain relevant for broader LLM-based systems, particularly in high-stakes domains such as clinical or pharmaceutical decision-support where incorrect outputs could lead to misleading interpretations.

Responsible deployment of LLM-based systems also requires appropriate governance and monitoring practices. Emerging LLMOps frameworks emphasize model documentation, evaluation protocols, and monitoring pipelines to detect drift or unintended behavior during deployment. Such governance mechanisms support transparency and accountability by enabling auditing, monitoring, and corrective interventions when unexpected outcomes occur in operational environments. Researchers and practitioners should therefore apply automated personality assessment systems responsibly and carefully consider ethical implications in real-world applications.

## Data Availability

The data analyzed in this study is subject to the following licenses/restrictions: data access requires a DUA and SPAN study approval. Requests to access these datasets should be directed to https://github.com/AI-for-Health-Data/OCEANprediction/tree/main/frontiers.

## References

[ref1] Alsini R. Naz A. Khan H. U. Bukhari A. Daud A. Ramzan M. (2024). Using deep learning and word embeddings for predicting human agreeableness behavior. Sci. Rep. 14:29875. doi: 10.1038/s41598-024-81506-8, 39622946 PMC11612277

[ref2] An S. Ma Z. Lin Z. Zheng N. Lou J.-G. Chen W. (2024). Make your llm fully utilize the context. Adv. Neural Inf. Proces. Syst. 37, 62160–62188. doi: 10.48550/arXiv.2404.16811

[ref3] Argamon S. Koppel M. Pennebaker J. W. Schler J. (2007). Mining the blogosphere: age, gender and the varieties of self-expression. First Monday. 12. doi: 10.5210/fm.v12i9.2003

[ref4] Bajestani S. S. Khalilzadeh M. M. Azarnoosh M. Kobravi H. R. (2024). Transentgat: a sentimentbased lexical psycholinguistic graph attention network for personality prediction. IEEE Access 12, 59630–59642. doi: 10.1109/ACCESS.2024.3390126

[ref5] Beltagy I. Peters M. E. Cohan A. (2020). Longformer: the long-document transformer. arXiv. doi: 10.48550/arXiv.2004.05150

[ref6] Berger J. Packard G. (2022). Using natural language processing to understand people and culture. Am. Psychol. 77, 525–537. doi: 10.1037/amp0000882 34914405

[ref7] Boyd R. L. Schwartz H. A. (2021). Natural language analysis and the psychology of verbal behavior: the past, present, and future states of the field. J. Lang. Soc. Psychol. 40, 21–41. doi: 10.1177/0261927X20967028, 34413563 PMC8373026

[ref8] Brown T. Mann B. Ryder N. Subbiah M. Kaplan J. D. Dhariwal P. . (2020). Language models are few-shot learners. Adv. Neural Inf. Proces. Syst. 33, 1877–1901. doi: 10.48550/arXiv.2005.14165

[ref9] Cao X. Kosinski M. (2024). Large language models know how the personality of public figures is perceived by the general public. Sci. Rep. 14:6735. doi: 10.1038/s41598-024-57271-z, 38509191 PMC10954708

[ref10] Costa P. T. McCrae R. R. (2008). “The revised neo personality inventory (neo-pi-r),” in The SAGE Handbook of Personality Theory and Assessment, eds. Boyle G. J. Matthews G. Saklofske D. H. (Thousand Oaks, CA: SAGE Publications Inc.) vol. 2, 179–198.

[ref11] Curtis B. Giorgi S. Buffone A. E. Ungar L. H. Ashford R. D. Hemmons J. . (2018). Can twitter be used to predict county excessive alcohol consumption rates? PLoS One 13:e0194290. doi: 10.1371/journal.pone.0194290, 29617408 PMC5884504

[ref12] Cutler A. Condon D. M. (2023). Deep lexical hypothesis: identifying personality structure in natural language. J. Pers. Soc. Psychol. 125, 173–197. doi: 10.1037/pspp0000443, 36395036

[ref13] Dai Z. Yang Z. Yang Y. Carbonell J. G. Le Q. Salakhutdinov R. (2019). Transformer-xl: Attentive Language Models beyond a Fixed-Length context. In Proceedings of the 57th Annual Meeting of the Association for Computational Linguistics. 2978–2988

[ref14] Danner M. Hadzic B. Gerhardt S. Ludwig S. Uslu I. Shao P. . (2023). Advancing mental health diagnostics: GPT-based method for depression detection. In 2023 62nd Annual Conference of the Society of Instrument and Control Engineers (SICE) (IEEE), 1290–1296

[ref15] De Bruyne L. Atanasova P. Augenstein I. (2022). Joint emotion label space modeling for affect lexica. Comput. Speech Lang. 71:101257. doi: 10.1016/j.csl.2021.101257

[ref16] Ding S. Shang J. Wang S. Sun Y. Tian H. Wu H. . (2021). Ernie-doc: a retrospective long-document modeling transformer. In Proceedings of the 59th Annual Meeting of the Association for Computational Linguistics and the 11th International Joint Conference on Natural Language Processing (Volume 1: Long Papers). 2914–2927

[ref17] Ding M. Zhou C. Yang H. Tang J. (2020). Cogltx: applying bert to long texts. Adv. Neural Inf. Proces. Syst. 33, 12792–12804.

[ref18] Eichstaedt J. C. Kern M. L. Yaden D. B. Schwartz H. A. Giorgi S. Park G. . (2021). Closed-and open-vocabulary approaches to text analysis: a review, quantitative comparison, and recommendations. Psychol. Methods 26, 398–427. doi: 10.1037/met0000349, 34726465

[ref19] Eichstaedt J. C. Smith R. J. Merchant R. M. Ungar L. H. Crutchley P. Preoţiuc-Pietro D. . (2018). Facebook language predicts depression in medical records. Proc. Natl. Acad. Sci. 115, 11203–11208. doi: 10.1073/pnas.1802331115, 30322910 PMC6217418

[ref20] Ganesan A.V. Lal Y.K. Nilsson A. Schwartz H.A., (2023). Systematic Evaluation of GPT-3 for Zero-Shot Personality Estimation.

[ref21] Geng B. Huan Z. Zhang X. He Y. Zhang L. Yuan F. . (2024). Breaking the Length barrier: Llm-Enhanced CTR Prediction in long Textual user Behaviors. In Proceedings of the 47th International ACM SIGIR Conference on Research and Development in Information Retrieval. 2311–2315

[ref22] Gong H. Shen Y. Yu D. Chen J. Yu D. (2020). Recurrent chunking mechanisms for long-text machine reading comprehension. In Proceedings of the 58th Annual Meeting of the Association for Computational Linguistics. 6751–6761

[ref23] Graziano W. G. Tobin R. M. (2017). “Agreeableness and the five factor model,” in The Oxford Handbook of the Five Factor Model, ed. Widiger T. A. (New York, NY: Oxford University Press) vol. 1, 105–131.

[ref24] Hatfield D. R. Ogles B. M. (2004). The use of outcome measures by psychologists in clinical practice. Prof. Psychol. Res. Pract. 35, 485–491. doi: 10.1037/0735-7028.35.5.485

[ref25] Hu E.J. Shen Y. Wallis P. Allen-Zhu Z. Li Y. Wang S. . (2021). Lora: Low-Rank Adaptation of Large Language Models.

[ref26] Huang Y. Xu J. Lai J. Jiang Z. Chen T. Li Z. . (2023). Advancing transformer architecture in long-context large language models: a comprehensive Survey. arXiv. doi: 10.48550/arXiv.2311.12351

[ref27] Iliev R. Dehghani M. Sagi E. (2015). Automated text analysis in psychology: methods, applications, and future developments. Lang. Cogn. 7, 265–290. doi: 10.1017/langcog.2014.30

[ref28] Jackson J. C. Watts J. List J.-M. Puryear C. Drabble R. Lindquist K. A. (2022). From text to thought: how analyzing language can advance psychological science. Perspect. Psychol. Sci. 17, 805–826. doi: 10.1177/17456916211004899, 34606730 PMC9069665

[ref29] Jackson J. J. Wood D. Bogg T. Walton K. E. Harms P. D. Roberts B. W. (2010). What do conscientious people do? Development and validation of the behavioral indicators of conscientiousness (BIC). J. Res. Pers. 44, 501–511. doi: 10.1016/j.jrp.2010.06.005, 21278818 PMC3028204

[ref30] Jain D. Kumar A. Beniwal R. (2022). Personality bert: a transformer-based model for personality detection from textual data. In Proceedings of International Conference on Computing and Communication Networks: ICCCN 2021 (Springer), 515–522

[ref31] John O. P. (2021). “History, measurement, and conceptual Elaboration of the big-five trait taxonomy,” in Handbook of Personality: Theory and Research, 4th ed. Eds. John O. P. Robins R. W. (New York, NY: The Guilford Press) 35–82.

[ref32] Joshy A. Sundar S. (2022). Analyzing the performance of sentiment analysis using BERT, DistilBERT, and RoBERTa. In 2022 IEEE International Power and Renewable Energy Conference (IPRECON). 1–6.

[ref33] Kjell O. Daukantaitė D. Sikström S. (2021). Computational language assessments of harmony¨ in life—not satisfaction with life or rating scales—correlate with cooperative behaviors. Front. Psychol. 12:601679. doi: 10.3389/fpsyg.2021.601679, 34045988 PMC8144476

[ref34] Kjell O. N. Sikstrom S. Kjell K. Schwartz H. A. (2022). Natural language analyzed with ai-based¨ transformers predict traditional subjective well-being measures approaching the theoretical upper limits in accuracy. Sci. Rep. 12:3918. doi: 10.1038/s41598-022-07520-w, 35273198 PMC8913644

[ref35] Kotov R. Gamez W. Schmidt F. Watson D. (2010). Linking “big” personality traits to anxiety, depressive, and substance use disorders: a meta-analysis. Psychol. Bull. 136, 768–821. doi: 10.1037/a0020327, 20804236

[ref36] Kotov R. Krueger R. F. Watson D. Achenbach T. M. Althoff R. R. Bagby R. M. . (2017). The hierarchical taxonomy of psychopathology (HiTOP): a dimensional alternative to traditional nosologies. J. Abnorm. Psychol. 126, 454–477. doi: 10.1037/abn0000258, 28333488

[ref37] Kwantes P. J. Derbentseva N. Lam Q. Vartanian O. Marmurek H. H. (2016). Assessing the big five personality traits with latent semantic analysis. Personal. Individ. Differ. 102, 229–233. doi: 10.1016/j.paid.2016.07.010

[ref38] Li J. Chen X. Hovy E. Jurafsky D. (2016). Visualizing and Understanding Neural Models in Nlp. In Proceedings of the 2016 Conference of the North American Chapter of the Association for Computational Linguistics: Human Language Technologies. 681–691

[ref39] Liao W. Zeng B. Yin X. Wei P. (2021). An improved aspect-category sentiment analysis model for text sentiment analysis based on RoBERTa. Appl. Intell. 51, 3522–3533. doi: 10.1007/s10489-020-01964-1

[ref40] Liu N. F. Lin K. Hewitt J. Paranjape A. Bevilacqua M. Petroni F. . (2024). Lost in the middle: how language models use long contexts. Trans. Assoc. Comput. Linguist. 12, 157–173. doi: 10.1162/tacl_a_00638

[ref41] Liu Y. Ott M. Goyal N. Du J. Joshi M. Chen D. . (2019). Roberta: a robustly optimized bert pretraining approach. arXiv. 20, 1218–1227. doi: 10.48550/arXiv.1907.11692

[ref42] Lukac M. (2024). Speech-based personality prediction using deep learning with acoustic and linguistic embeddings. Sci. Rep. 14:30149. doi: 10.1038/s41598-024-81047-0, 39627367 PMC11615297

[ref43] Lynn V. Balasubramanian N. Schwartz H. A. (2020). Hierarchical modeling for user personality prediction: the role of message-level attention. In Proceedings of the 58th Annual Meeting of the Association for Computational Linguistics. 5306–5316

[ref44] MacAvaney S. Mittu A. Coppersmith G. Leintz J. Resnik P. (2021). Community-level research on suicidality prediction in a secure environment: overview of the Clpsych 2021 shared task. In Proceedings of the Seventh Workshop on Computational Linguistics and Clinical Psychology: Improving Access. 70–80

[ref45] Mehta Y. Fatehi S. Kazameini A. Stachl C. Cambria E. Eetemadi S. (2020). Bottom-up and top-down: predicting personality with psycholinguistic and language model features. In 2020 IEEE International Conference on data mining (ICDM) (IEEE), 1184–1189

[ref46] Oltmanns J. R. Jackson J. J. Oltmanns T. F. (2020). Personality change: longitudinal self-other agreement and convergence with retrospective-reports. J. Pers. Soc. Psychol. 118, 1065–1079. doi: 10.1037/pspp0000238, 30843725 PMC6732056

[ref47] Ozer D. J. Benet-Martinez V. (2006). Personality and the prediction of consequential outcomes. Annu. Rev. Psychol. 57, 401–421. doi: 10.1146/annurev.psych.57.102904.190127 16318601

[ref48] Pappagari R. Zelasko P. Villalba J. Carmiel Y. Dehak N. (2019). Hierarchical transformers for long document classification. In 2019 IEEE Automatic speech Recognition and Understanding Workshop (ASRU) (IEEE), 838–844

[ref49] Park G. Schwartz H. A. Eichstaedt J. C. Kern M. L. Kosinski M. Stillwell D. J. . (2015). Automatic personality assessment through social media language. J. Pers. Soc. Psychol. 108, 934–952. doi: 10.1037/pspp0000020, 25365036

[ref50] Paulhus D. L. Vazire S. (2007). “The self-report method,” in Handbook of Research Methods in Personality Psychology, eds. Robins R. W. Fraley R. C. Krueger R. F. (New York: Guilford Press), 224–239.

[ref51] Peters H. Matz S. C. Gelfand M. . (2024). Large language models can infer psychological dispositions of social media users. PNAS Nexus 3:pgae231. doi: 10.1093/pnasnexus/pgae231, 38948324 PMC11211928

[ref52] Piastra M. Catellani P. (2025). On the emergent capabilities of chatgpt 4 to estimate personality traits. Front. Artif. Intell. 8:1484260. doi: 10.3389/frai.2025.1484260, 40017486 PMC11865037

[ref53] Piotrowski C. (1999). Assessment practices in the era of managed care: current status and future directions. J. Clin. Psychol. 55, 787–796. doi: 10.1002/(SICI)1097-4679(199907)55:7<787::AID-JCLP2>3.0.CO;2-U, 10866016

[ref54] Rae J. W. Potapenko A. Jayakumar S. M. Lillicrap T. P. (2019). Compressive transformers for long-range sequence modelling. arXiv. doi: 10.48550/arXiv.1911.05507

[ref55] Schwaba T. (2019). “The structure, measurement, and development of openness to experience across adulthood,” in Handbook of Personality Development, eds. McAdams D. P. Shiner R. L. Tackett J. L. (New York, NY: The Guilford Press) 185–200.

[ref56] Schwartz H. A. Eichstaedt J. C. Kern M. L. Dziurzynski L. Ramones S. M. Agrawal M. . (2013). Personality, gender, and age in the language of social media: the open-vocabulary approach. PLoS One 8:e73791. doi: 10.1371/journal.pone.0073791, 24086296 PMC3783449

[ref57] Schwartz H. A. Ungar L. H. (2015). Data-driven content analysis of social media: a systematic overview of automated methods. Ann. Am. Acad. Pol. Soc. Sci. 659, 78–94. doi: 10.1177/0002716215569197

[ref58] Sendas N. Rajale D. (2024). “Future trends in MLOps,” in The Definitive Guide to Machine Learning Operations in AWS: Machine Learning Scalability and Optimization with AWS, (Berkeley, CA: Springer), 371–423.

[ref59] Senn S. Tlachac M. Flores R. Rundensteiner E. (2022). Ensembles of bert for depression classification. In 2022 44th Annual International Conference of the IEEE Engineering in Medicine & Biology Society (EMBC) (IEEE), 4691–4694 10.1109/EMBC48229.2022.9871120 36085764

[ref60] Simchon A. Sutton A. Edwards M. Lewandowsky S. (2023). Online reading habits can reveal personality traits: towards detecting psychological microtargeting. PNAS Nexus 2:pgad191. doi: 10.1093/pnasnexus/pgad191, 37333766 PMC10276193

[ref61] Soto C. J. (2019). How replicable are links between personality traits and consequential life outcomes? The life outcomes of personality replication project. Psychol. Sci. 30, 711–727. doi: 10.1177/0956797619831612, 30950321

[ref62] Sun J. Schwartz H. A. Son Y. Kern M. L. Vazire S. (2020). The language of well-being tracking fluctuations in emotion experience through everyday speech. J. Pers. Soc. Psychol. 118, 364–387. doi: 10.1037/pspp0000244 30945904

[ref63] Tausczik Y. R. Pennebaker J. W. (2010). The psychological meaning of words: LIWC and computerized text analysis methods. J. Lang. Soc. Psychol. 29, 24–54. doi: 10.1177/0261927X09351676

[ref64] Wang Z. Ng P. Ma X. Nallapati R. Xiang B. (2019). Multi-passage bert: a globally normalized bert model for open-domain question answering. In Proceedings of the 2019 Conference on Empirical Methods in natural Language Processing and the 9th International Joint Conference on natural Language Processing (EMNLP-IJCNLP). 5878–5882

[ref65] Widiger T. A. Oltmanns J. R. (2017). Neuroticism is a fundamental domain of personality with enormous public health implications. World Psychiatry 16, 144–145. doi: 10.1002/wps.20411, 28498583 PMC5428182

[ref66] Wright C. V. Beattie S. G. Galper D. I. Church A. S. Bufka L. F. Brabender V. M. . (2017). Assessment practices of professional psychologists: results of a national survey. Prof. Psychol. Res. Pract. 48, 73–78. doi: 10.1037/pro0000086

[ref67] Wright A. G. Ringwald W. R. Vize C. E. Eichstaedt J. C. Angstadt M. Taxali A. . (2026). Assessing personality using zero-shot generative ai scoring of brief open-ended text. Nat. Hum. Behav. 10, 541–555. doi: 10.1038/s41562-025-02389-x 41617861 PMC12974486

[ref68] Wright A. J. Weston S. J. Norton S. Voss M. Bogdan R. Oltmanns T. F. . (2022). Prospective self-and informant-personality associations with inflammation, health behaviors, and health indicators. Health Psychol. 41, 121–133. doi: 10.1037/hea0001162, 35238582 PMC9775638

[ref69] Wu Q. Lan Z. Gu J. Yu Z. (2020). Memformer: the memory-augmented transformer. arXiv. 2022, 308–318. doi: 10.48550/arXiv.2010.06891

[ref70] Zhang R. Wei Z. Shi Y. Chen Y. (2020). Bert-al: Bert for Arbitrarily Long Document Understanding.

[ref71] Zhu J. Li X. Maharjan J. Coifman K. G. Jin R. (2025). Benchmarking personality inference in large language models using real-world conversations. J. Psychiatry Brain Sci. 10:e250020. doi: 10.20900/jpbs.20250020

